# Supplementary material for: Smoking-associated gene expression alterations in nasal epithelium reveal immune impairment linked to lung cancer risk
Source: Genome Med. 2024 Apr 8;16:54. doi: 10.1186/s13073-024-01317-4 (PMC11000304; doi:10.1186/s13073-024-01317-4)
Supplement: Supplementary file 1 — Additional file 1: Supplementary Figures S1-S16. [file 13073_2024_1317_MOESM1_ESM.pdf]

Supplementary Figures

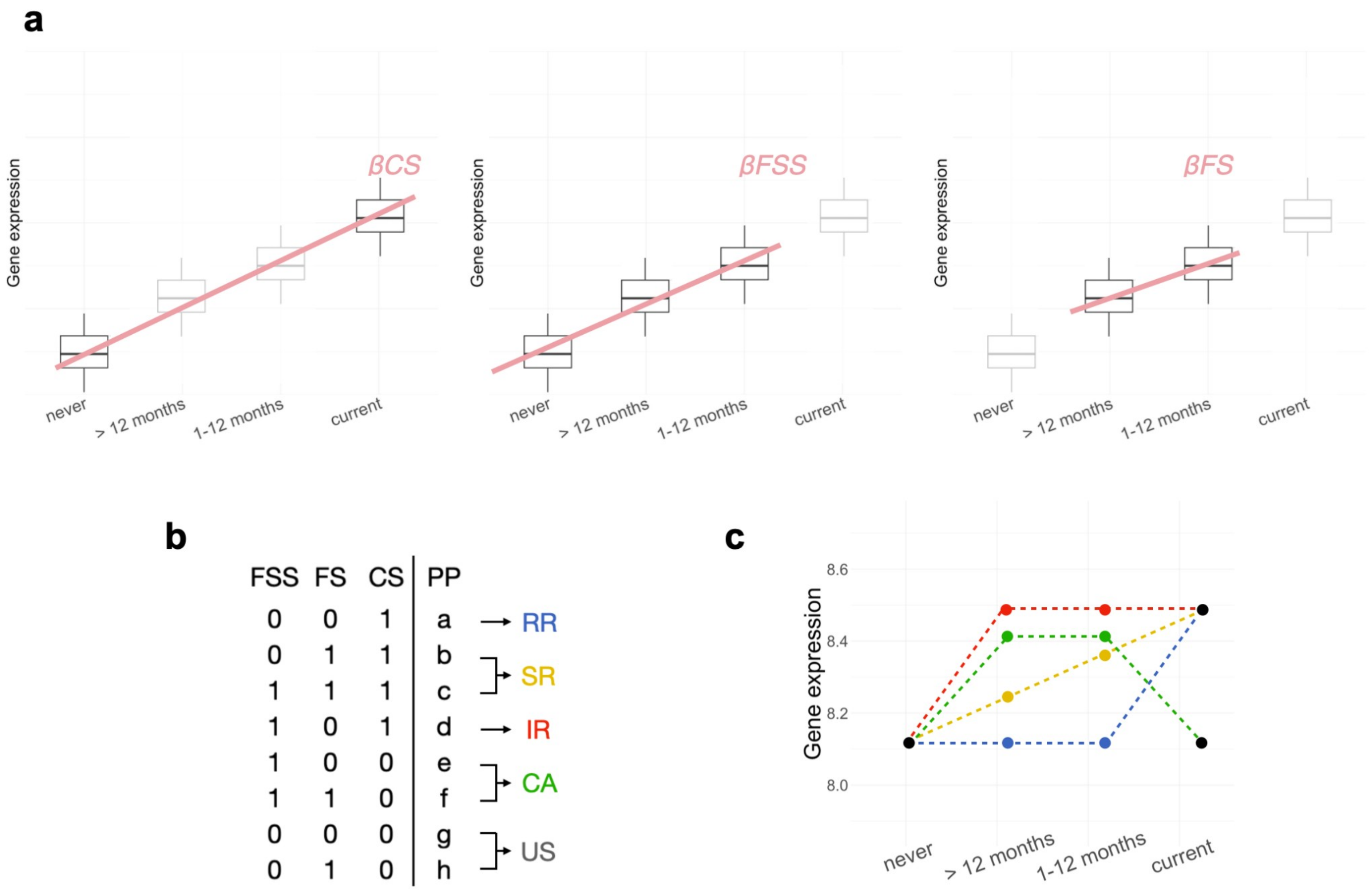

Fig. S1 Smoke injury reversibility analysis. (**a)** The slope coefficients associated to the three smoking status variables included in the Bayesian model (CS: current smoker status, FSS: former smoker status, FS: former smoker's time since quit). **(b)** Description of model selection procedure used to assign each gene to a reversibility class: the table shows all possible combinations of inclusion/exclusion of the three smoking status variables. **(c)** In blue, yellow and red, schematic of a gene with altered expression in current compared to never smokers, and the three possible trajectories after smoking cessation, corresponding to the RR, SR and IR reversibility classes; in green, schematic of a gene with no expression different in current versus never smokers, but altered expression in former smokers, corresponding to the CA class.
US: not affected; RR: rapidly reversible (blue) SR: slowly reversible (yellow); IR:irreversible (red); CA: cessation-associated (green).

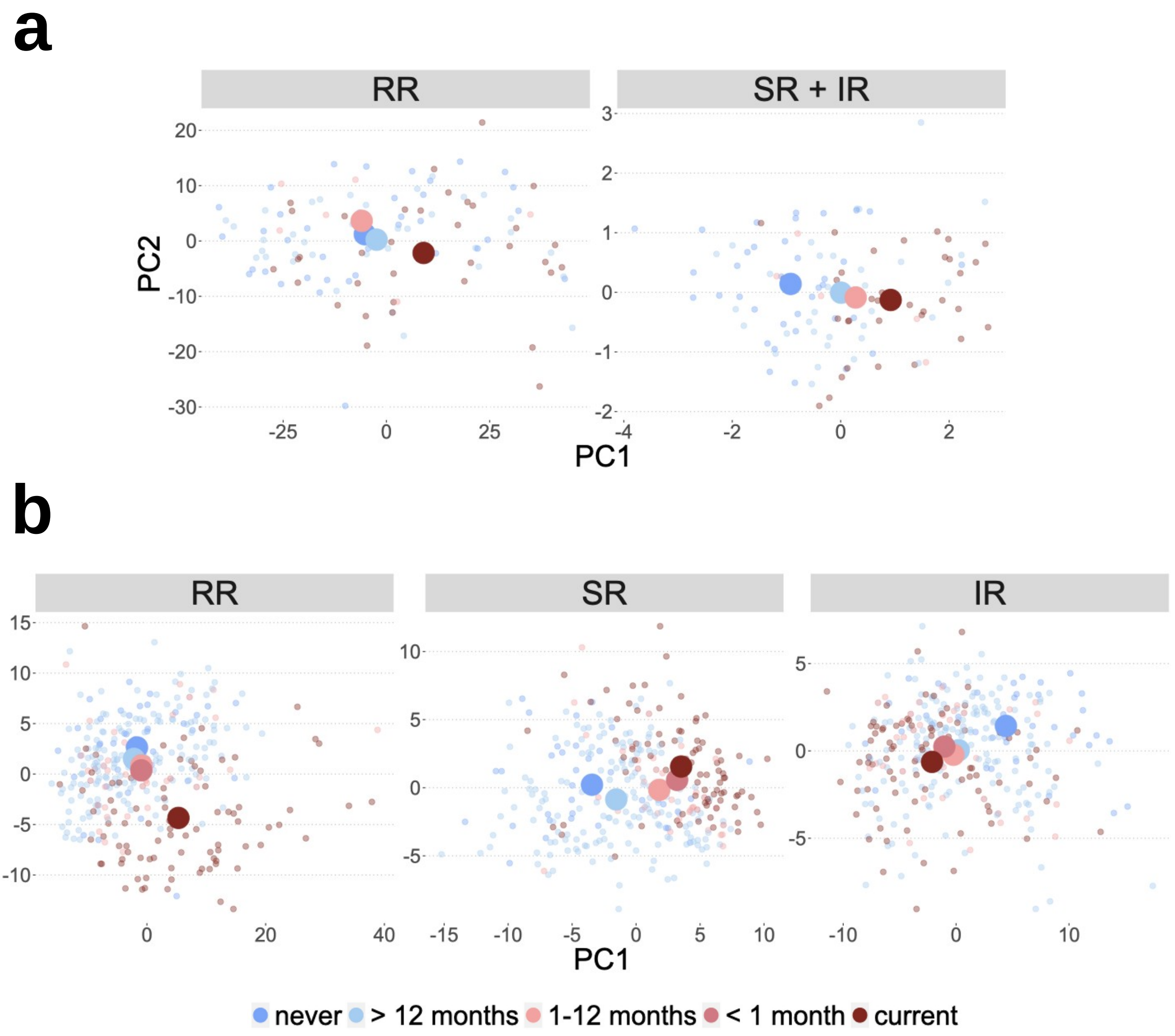

Fig. S2 Principal component analysis on the genes belonging to different reversibility classes. RR: Rapidly reversible genes, SR: Slowly reversible genes; IR:irreversible genes. Each small dot is a patient and colors indicate the smoking status of the patient. Large dots represent the mean of all patients for each smoking class. **(a):** nasal samples from healthy volunteers, using the reversibility classes from the bayesian model on the healthy volunteer group. Since only 2 genes were classified as IR, PCA was performed jointly for SR and IR genes **(b):** nasal samples from clinic subjects (cancer + benign), using the reversibility classes from the bayesian model on the clinic group.

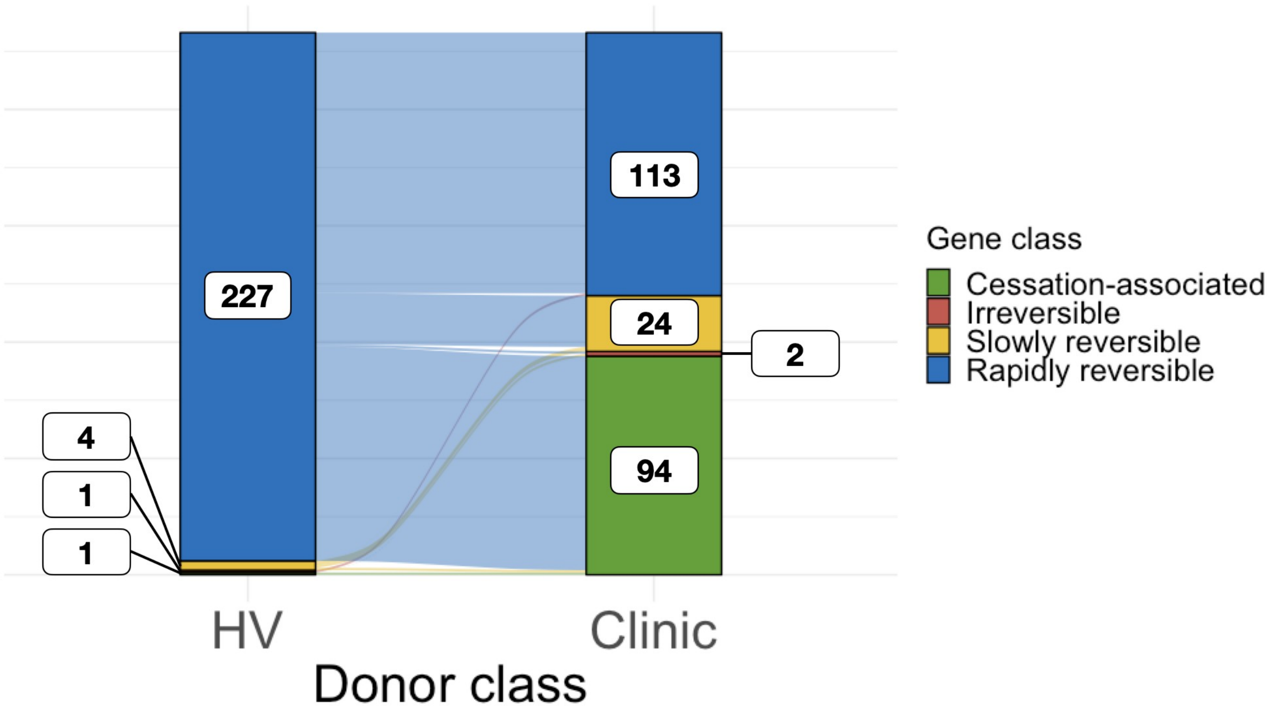

Fig. S3 Smoke injury reversibility. Comparison of the reversibility dynamics in the genes identified as affected by smoking both in healthy volunteers and clinic patients (n=233). Plot is showing the reversibility dynamics in the healthy volunteer (left) and clinic (right) donor groups. Color bars represent the number of genes in each reversibility class (blue: Rapidly reversible, yellow: slowly reversible, red: irreversible, green: cessation associated).

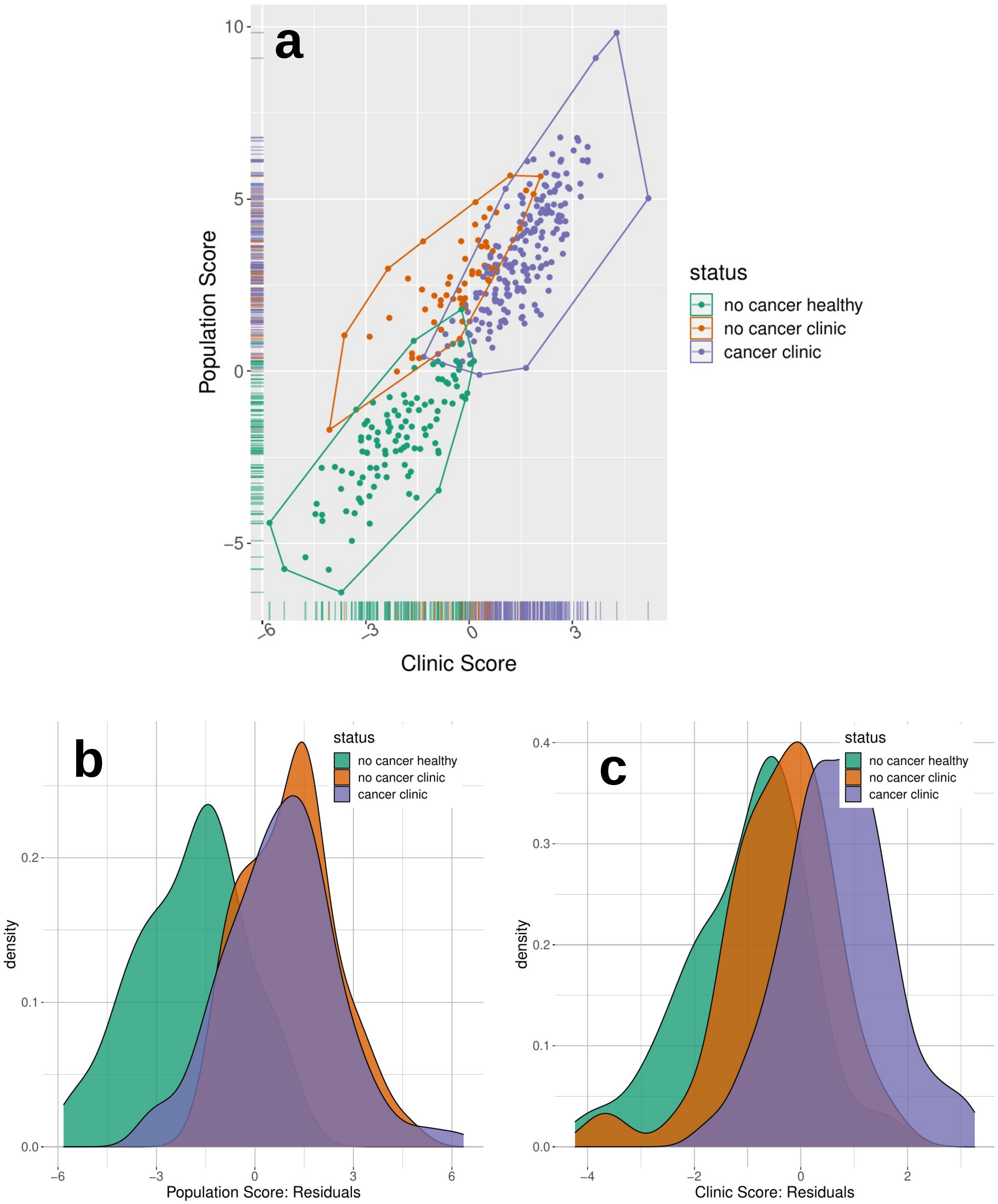

Fig. S4: Population and clinic risk scores. (a) Correlation between the clinic risk score and the population risk score for each patient. Each dot represent a single patient (green: healthy volunteer; orange: clinic benign; purple: clinic cancer). **(b-c)**: Distribution of the risk scores after removing the effect attributed to clinical informations for the population risk score **(b)** and the clinic risk score **(c)**.

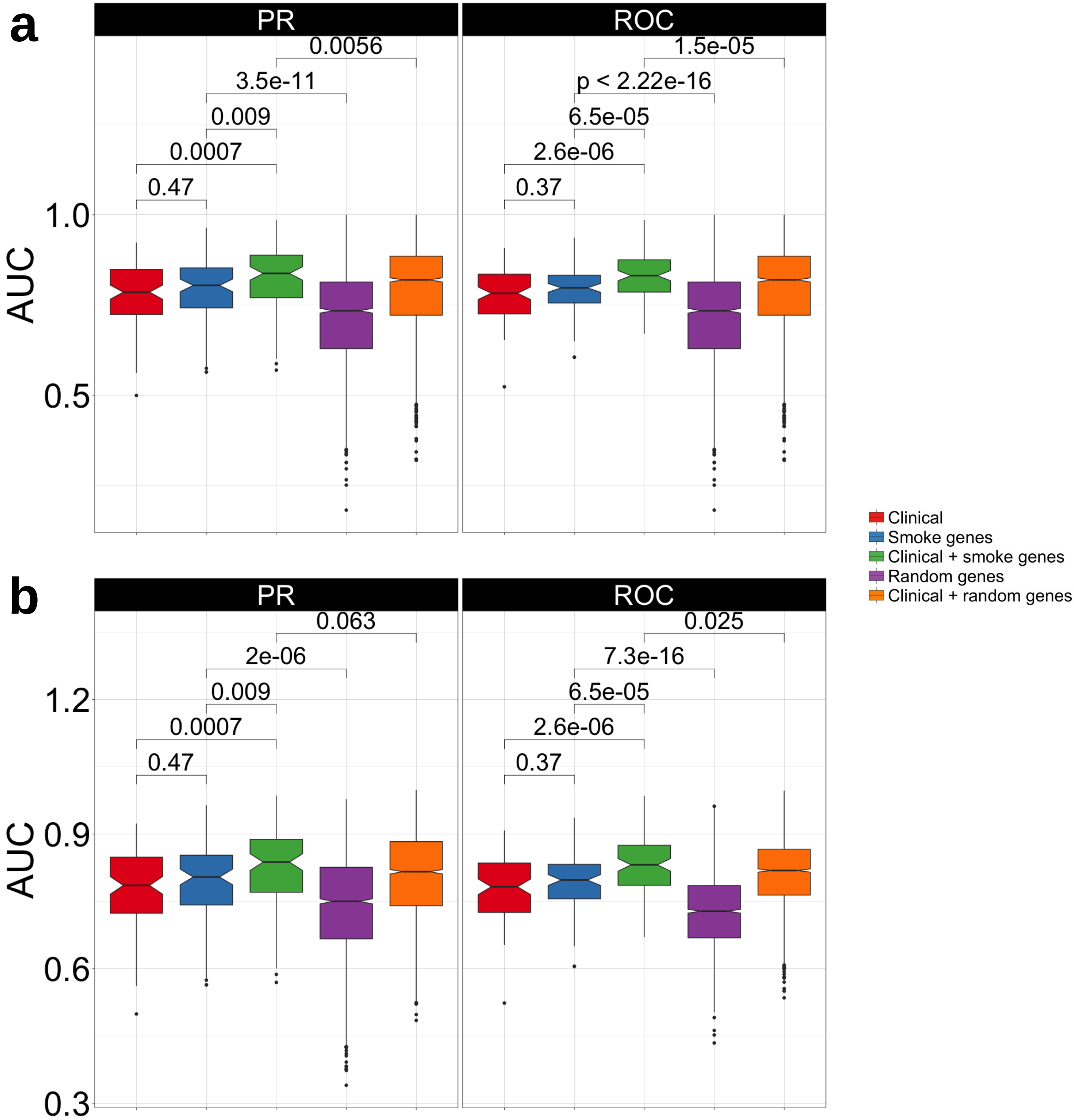

Fig. S5: Area under the Curve (AUC) calculated after cross validation (10 times 10-fold CV) for different models. Clinical: model trained on clinical data only, Smoke genes: model trained on the expression of smoke response genes only, Clinical + smoke genes: model trained on clinical data and expression of smoke response genes, Random genes: model trained on expression of a set of randomly selected genes, Clinical + random genes: model trained on clinical data and expression of a set of randomly selected genes. **(a)** Area under the PR and ROC curve for the population score, **(b)** Area under the PR and ROC curve for the clinic score.

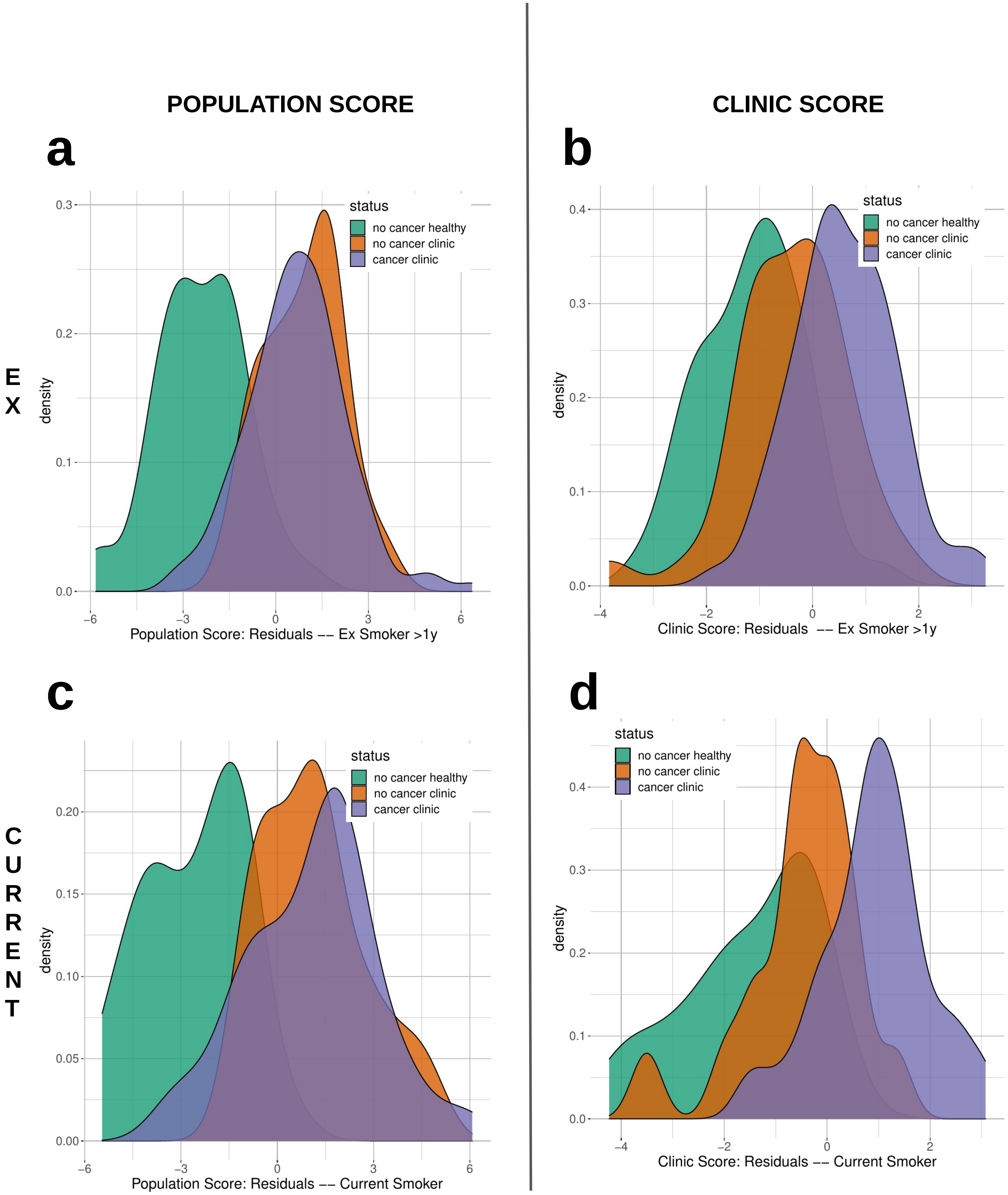

Fig. S6: Risk scores stratified by smoking status. Distribution of the population **(left)** and clinic **(right)** risk scores in ex smokers who stopped smoking for more than 1 year (**a-b**) and in current smokers (**c-d**), after removing the effect attributed to clinical covariates.

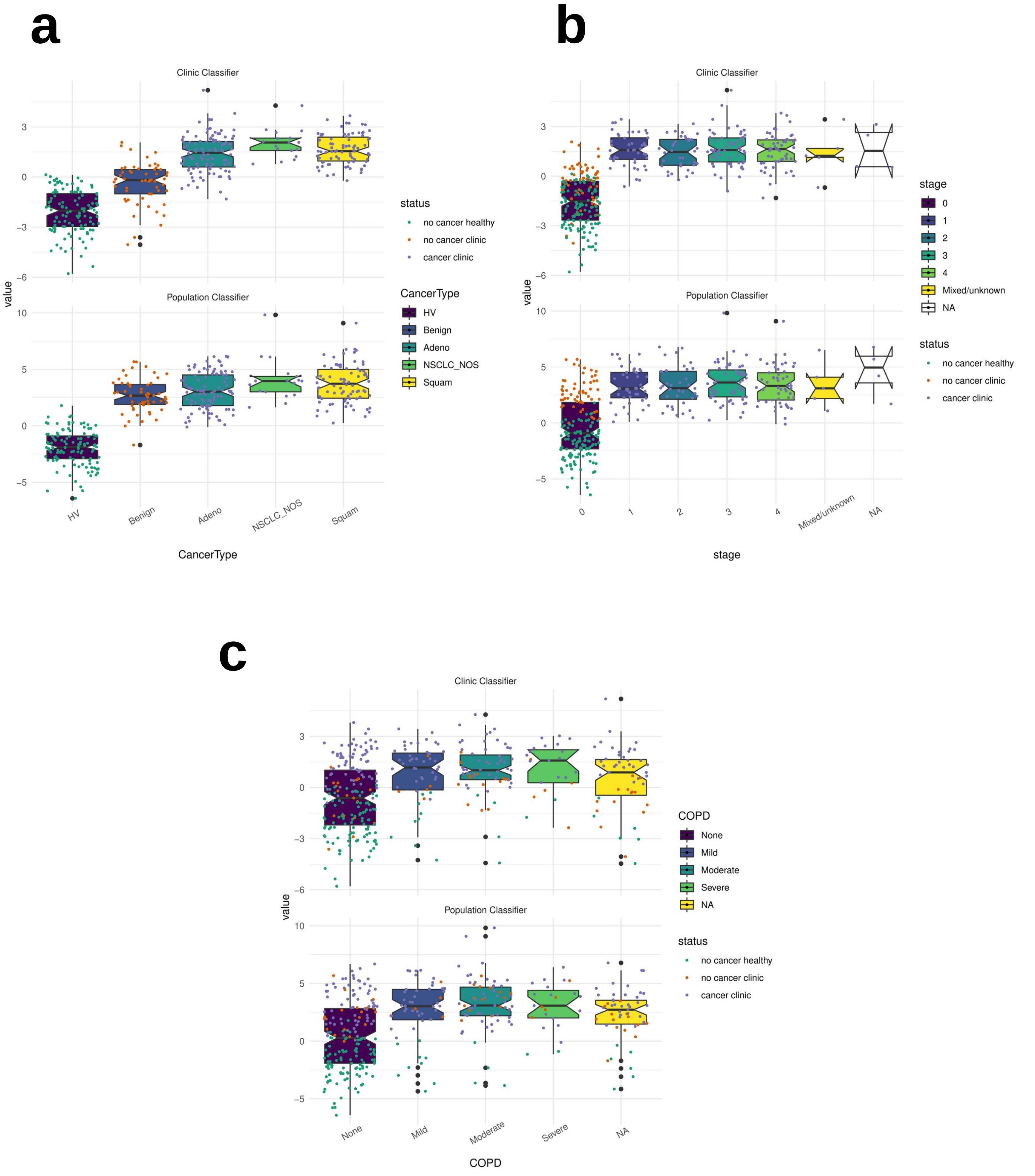

Fig. S7: Clinic and population scores stratified for different clinical variables. Distribution of the clinic (**Top rows)** and population **(bottom rows)** risk score in subjects depending on (**a**) The type of cancer (**b**) the stage of the cancer (**c**) the COPD status. Color of the dot indicate for each individual subject his status, namely healthy volunteer (green), clinic benign (orange) or clinic cancer (purple)

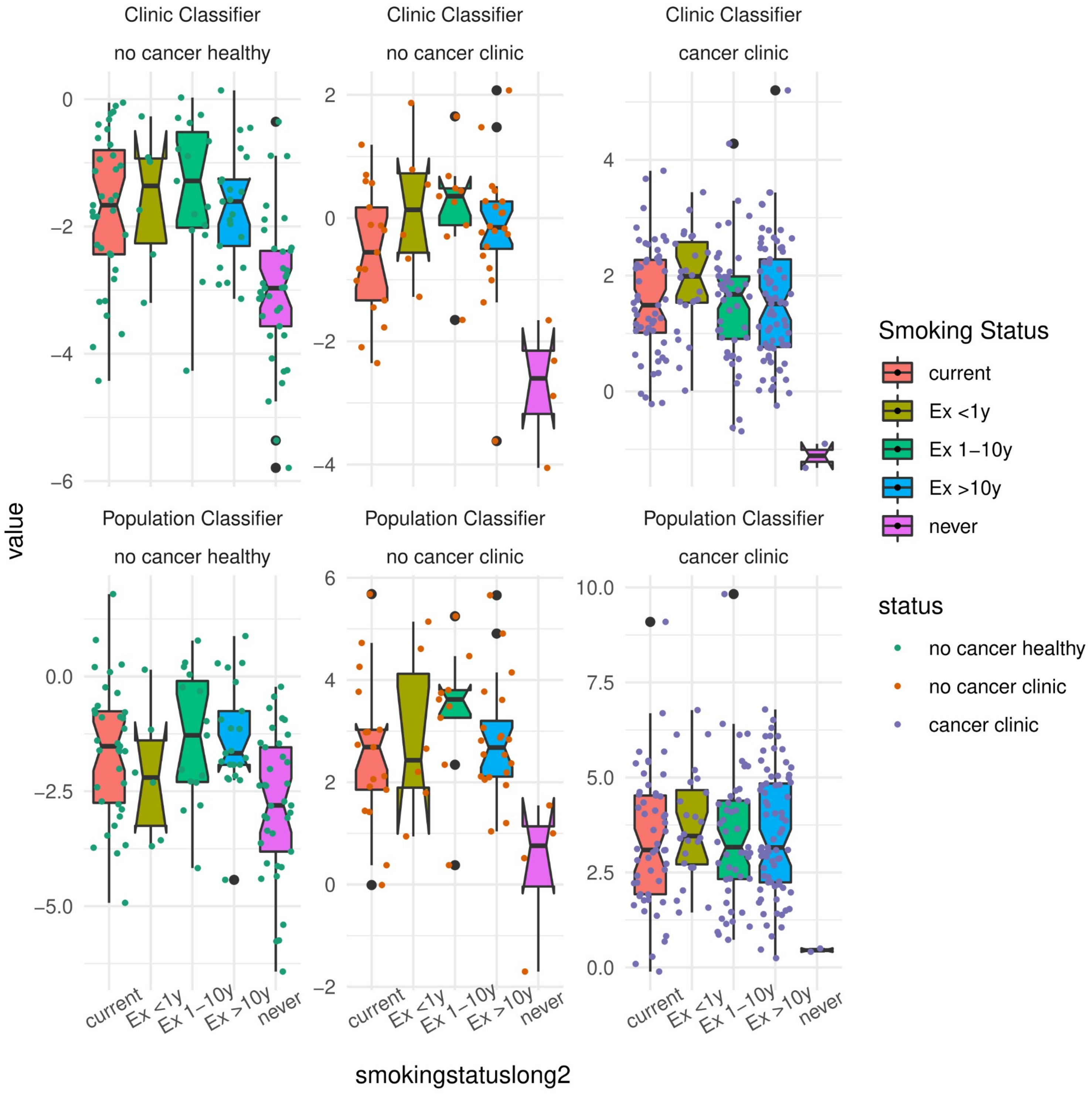

Fig. S8: Clinic and population scores depending on smoking status: Distribution of the clinic (**Top row)** and population **(bottom row)** risk score in subjects depending on their smoking status. Scores are represented separately for healthy volunteers (left), clinic benign (middle) and clinic cancer (right). Color of the dot indicate for each individual subject their status, namely healthy volunteer (green), clinic benign (orange) or clinic cancer (purple)

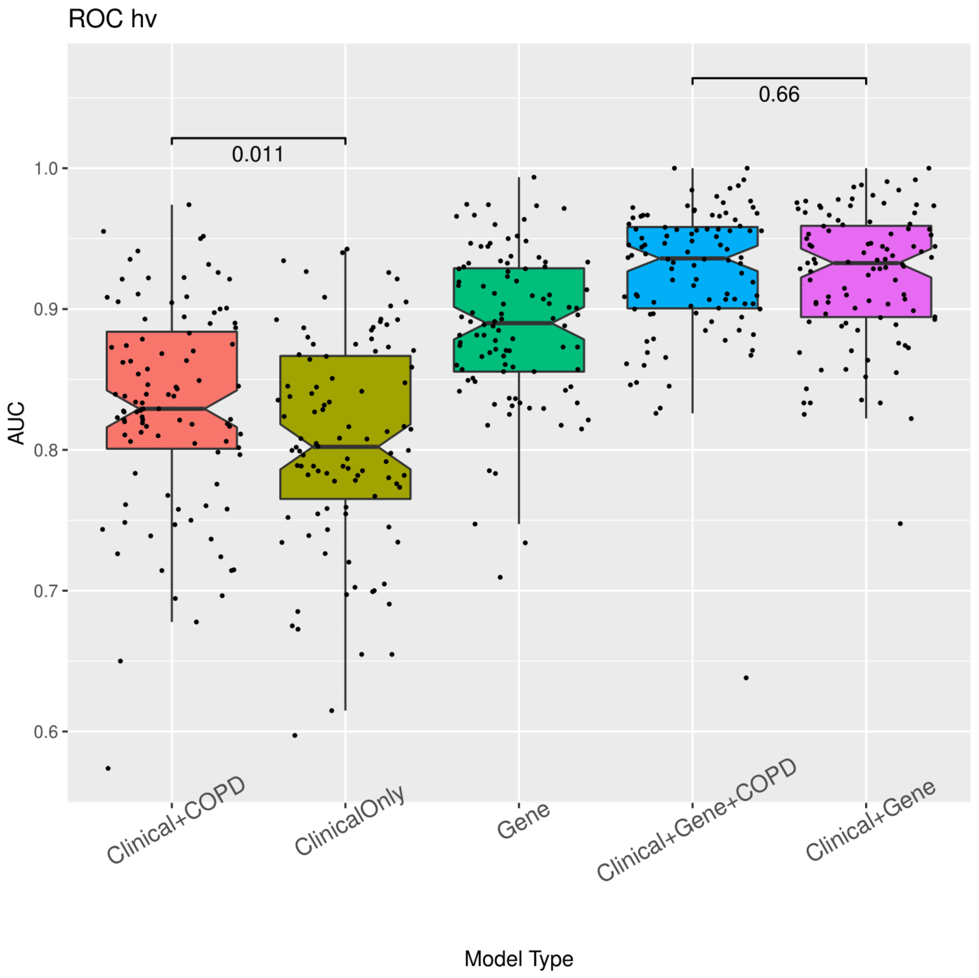

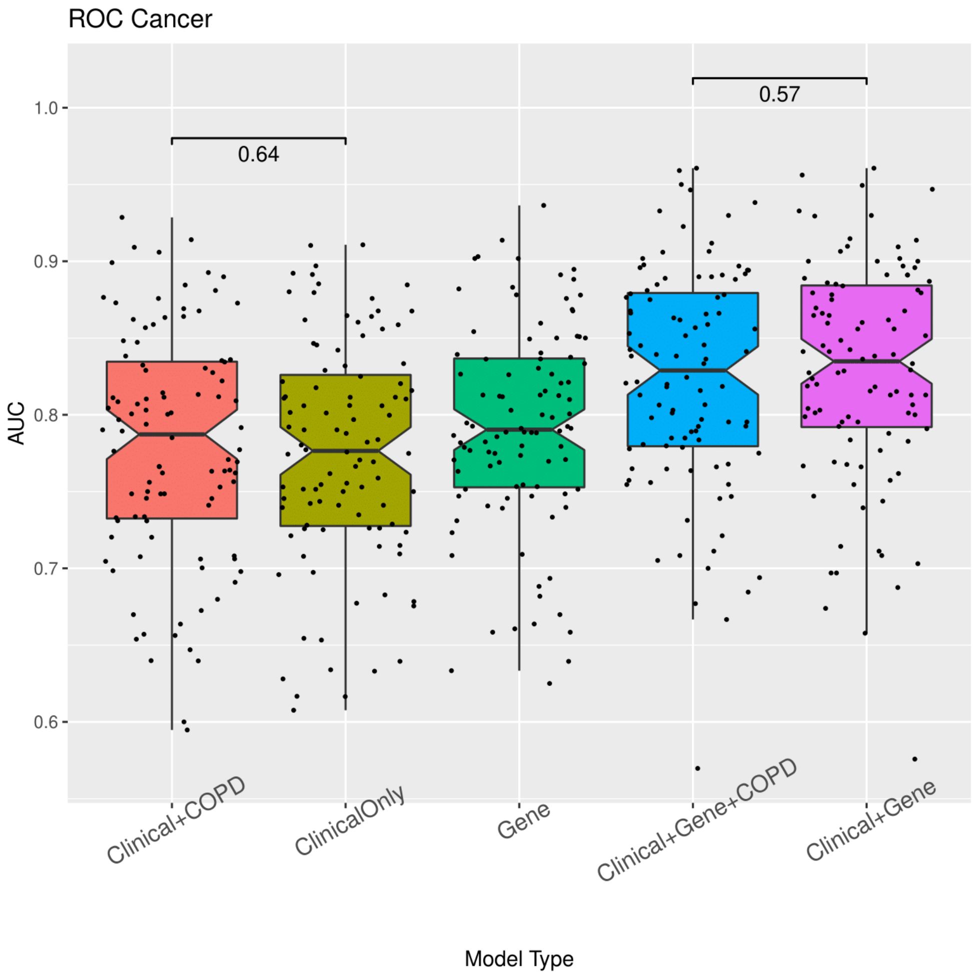

Fig. S9: Effect of COPD on the population (top) and clinic (bottom) classifiers. Area under the ROC curve, in 100 CV rounds, for a clinical-only model including COPD (red), a clinical-only model without COPD (brown) the model constructed on the response genes (green), a model constructed on a combination of clinical information *including* COPD (blue) a model constructed on a combination of clinical information *without* COPD and response genes (purple) for the population **(top)** and clinic **(bottom)** classifiers. P-values given above each box are computed using a 2 sample t-test, to test the difference between the models with and without COPD

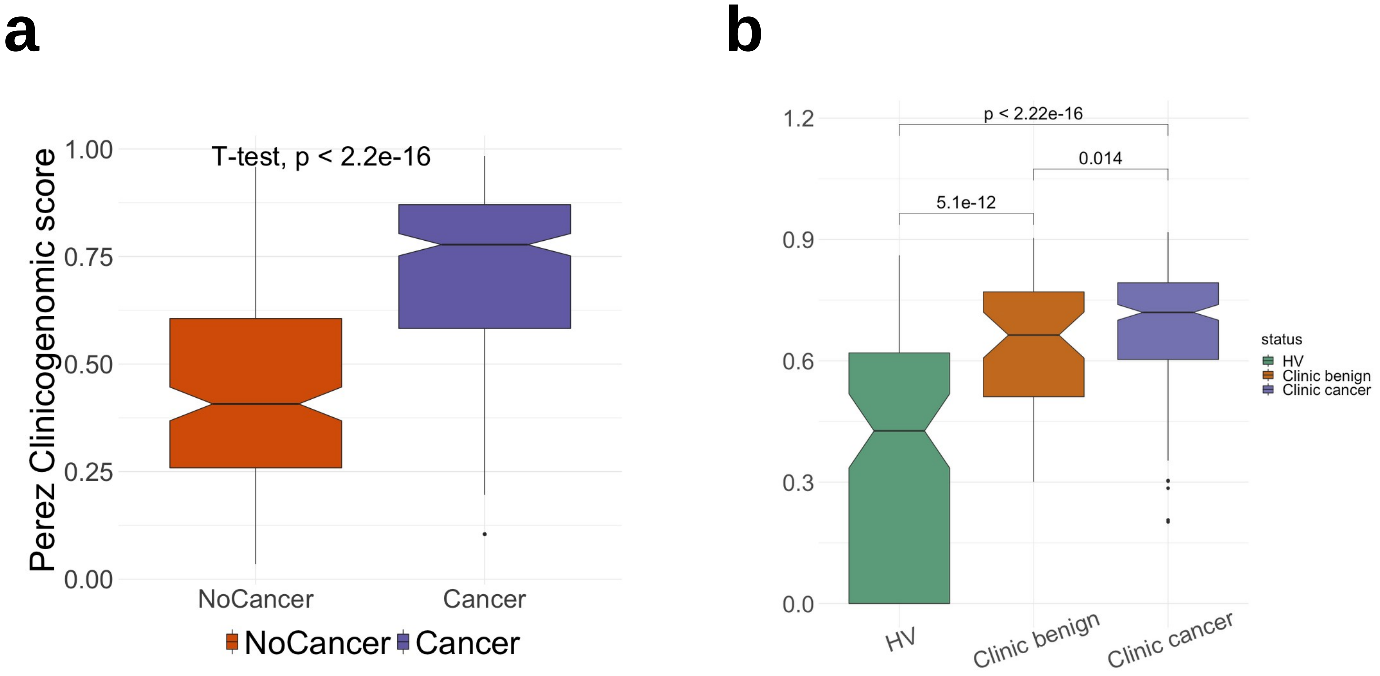

Fig. S10: Comparison with classifier from Perez-Rogers et al. (2017). (a) Perez-Rogers' clinico-genomic model applied to the AEGIS nasal cohort for patient with (purple) or without (orange) cancer. **(b)** Perez-Rogers' clinico-genomic model applied to nasal samples from our cohort, represented separately for healthy volunteers (green), patient with (purple) or without (orange) cancer. P-values were calculated with two-samples t-tests.

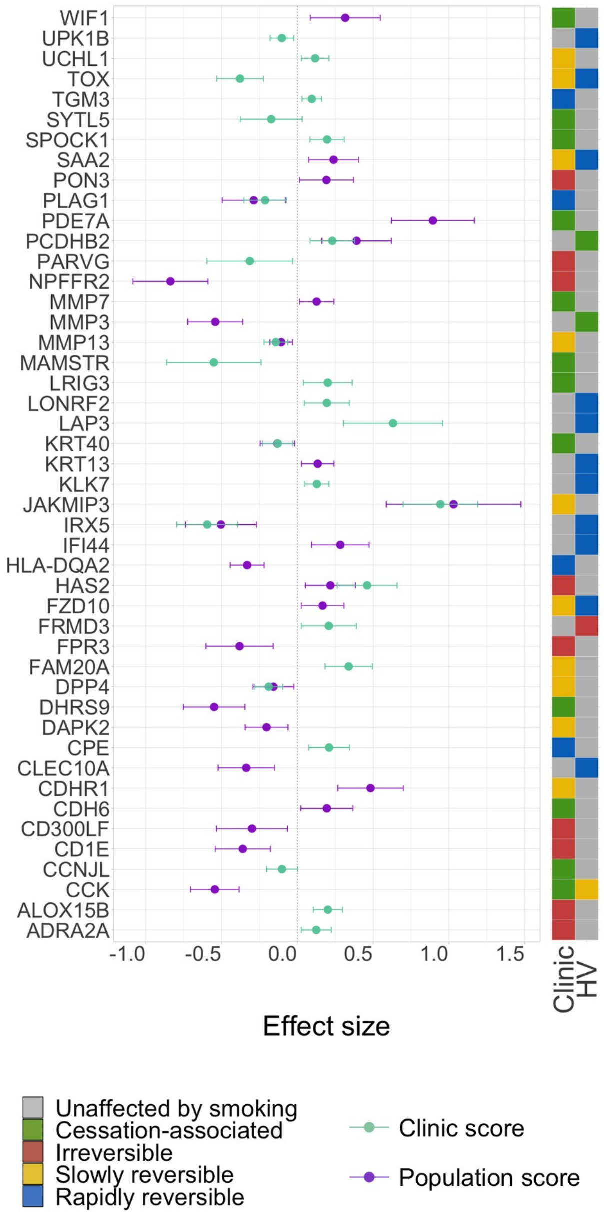

Fig. S11: Genes robustly contributing to the population and clinic risk scores. The weight of the genes selected in more than 80% of cross validations in the population (purple dot) and clinic (light green dot) classifiers. The plot presents the mean weight of each gene over all cross validations and the error bars represent standard deviation; the annotation track on the right shows the reversibility classes of the genes in the clinic groups (left column) and in the healthy volunteers (right column). grey: not affected by smoke; blue: rapidly reversible, yellow: slowly reversible, red: irreversible; green : cessation-associated. When the gene is not robustly contributing (*i.e.* is selected in less than 80% of the cross validation experiments) in both models, we present its weight in one model only.

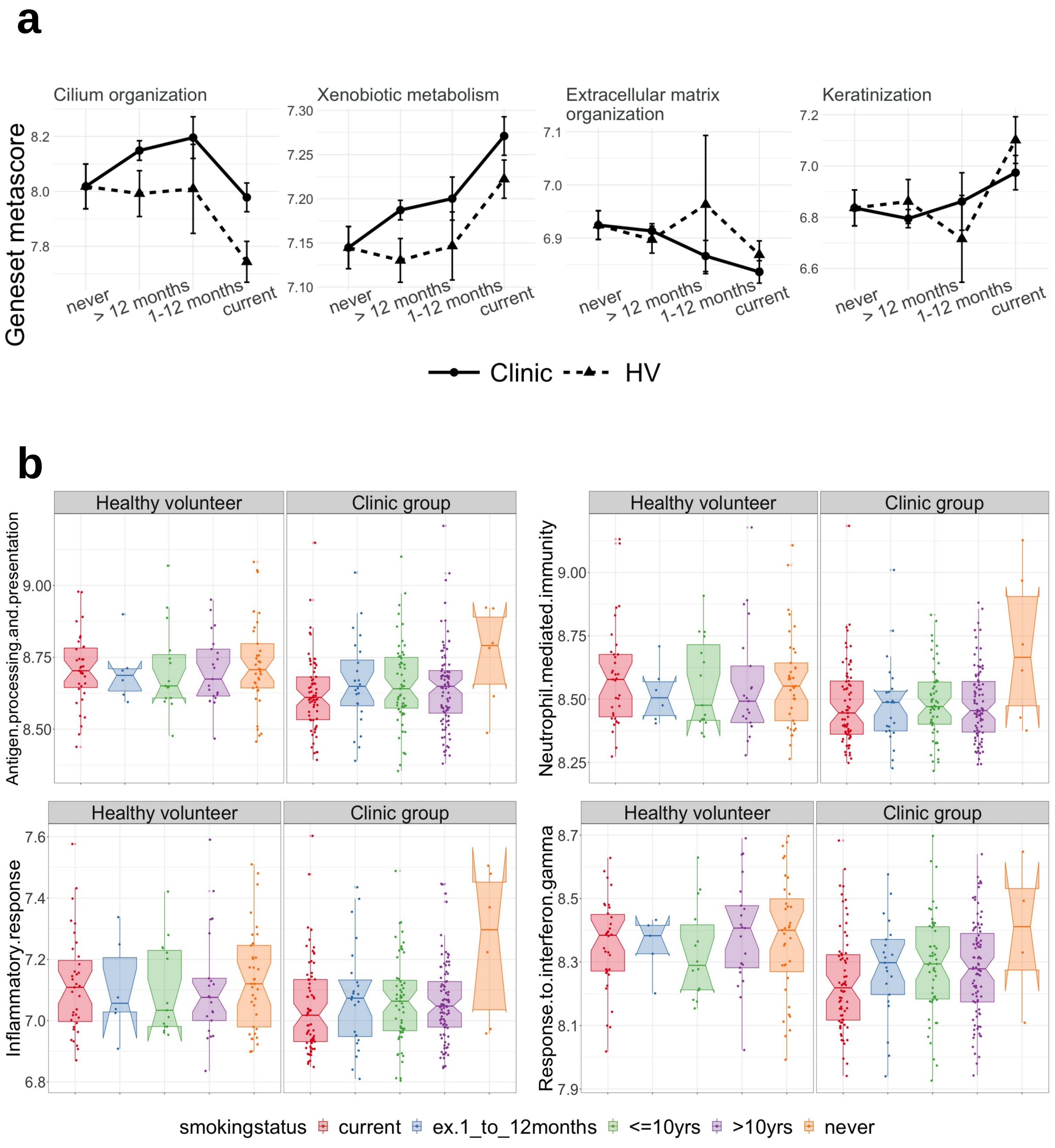

Fig. S12 Pathway metascore depending on the smoking status. (a) Differences in pathways' metascores response to smoke in clinic patients (plain line, circle dot) or in healthy volunteers (dotted line, triangle dot) for four GO terms involved in smoke injury response in nasal epithelium ; **(b)** Differences in pathways' metascores for immune-related genesets in healthy volunteers (left panels) and in the clinic group (right panels) . Former smokers with time since quit > 1 year were divided into former smokers who quit <= 10 years and >10 years before sample collection. Geneset metascores were calculated by averaging the nasal expression of genes belonging to each GO term. Red: Current smokers, blue: ex smokers between 1 and 12 months after smoking cessation, green: ex smokers between 1 and years after smoking cessation, purple: ex smokers over 10 years after smoking cessation, orange: never smokers.

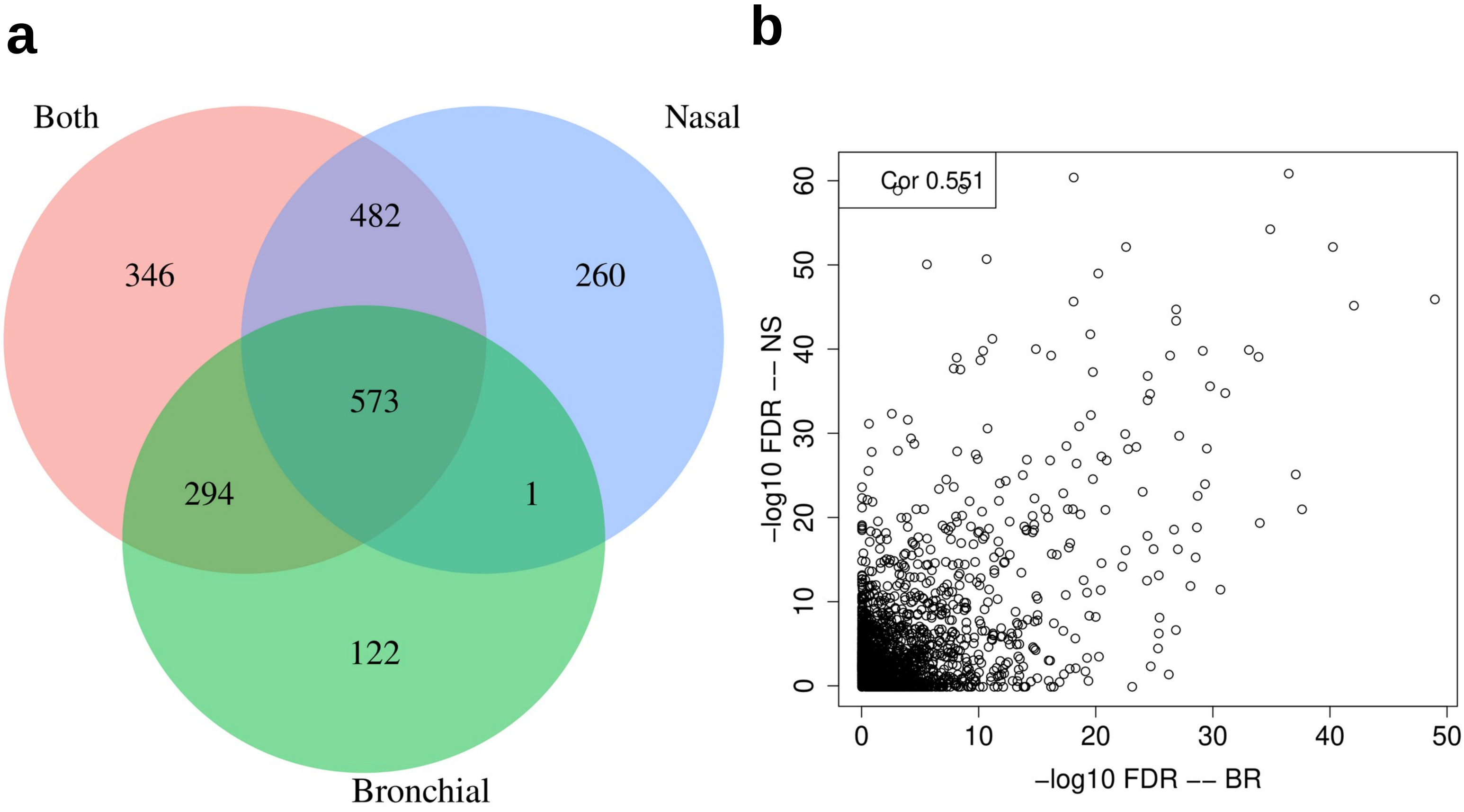

Fig. S13: Overlap of eQTL Analysis. eQTLs in the nasal and bronchial tissue are strongly correlated. **a:** A venn diagram of the eQTL genes found in the nasal tissue (blue), in the bronchial tissues (green) or in an analysis conducted jointly in the two tissued (red). **b:** Correlation between the corrected (see method for details on the multiple correction procedure) p-values (-log10) of the eQTL test in all tested genes (one dot per gene) between the test conducted in the bronchial (BR) and nasal (NS) tissues. The number in the top left corner represent the spearman correlation values between the corrected p-values in the nasal samples and in the bronchial samples.

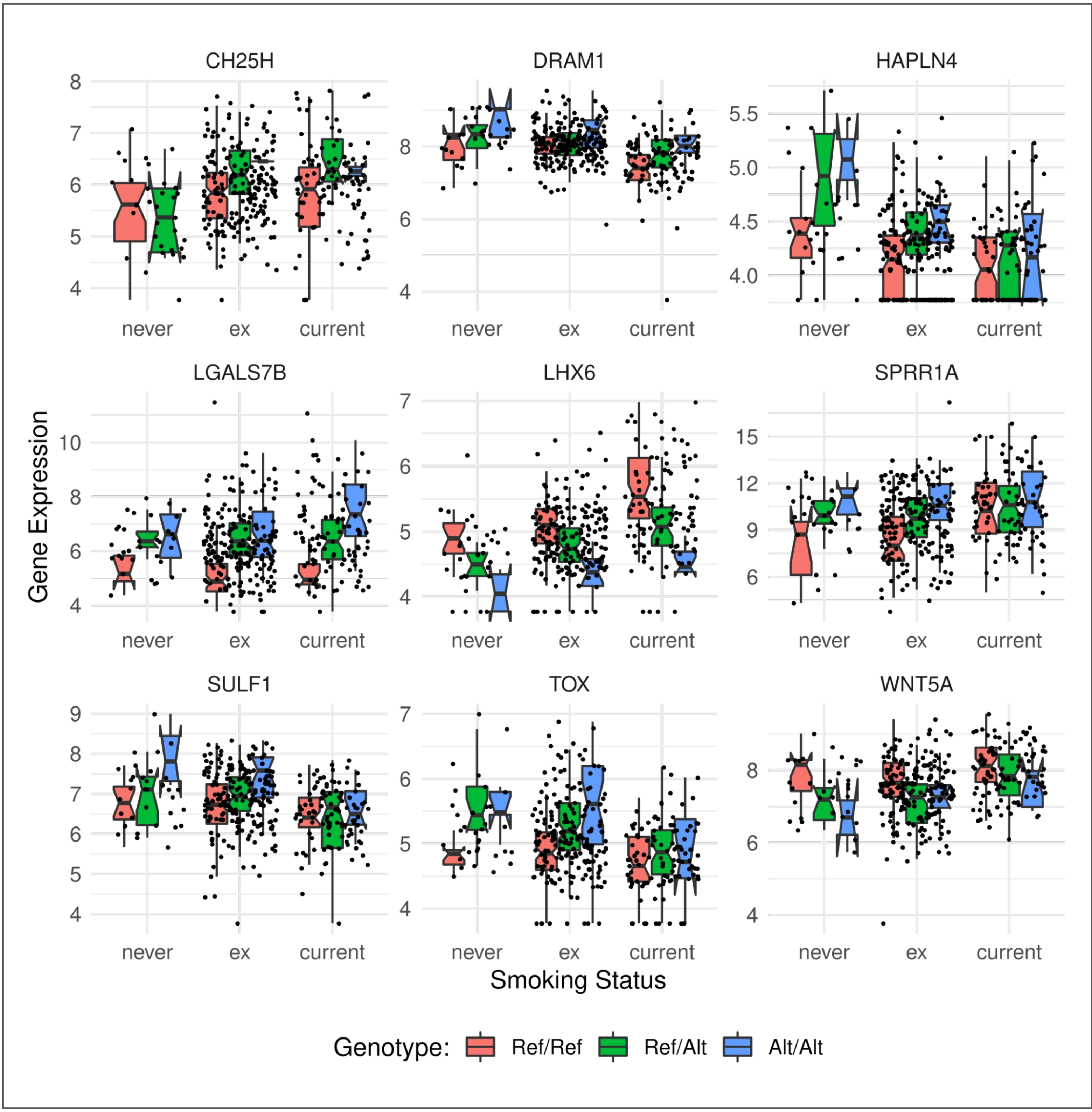

Fig. S14: Combined environmental and genetic effect on gene expression in nasal tissues. Plots are shown for 9/10 genes with a significant interaction effect between smoking status and the genotype of the patient at the lead eQTL position (one gene shown in main text), we present the expression level of the gene separately for never, former and current smokers. Samples are further stratified depending on the genotype of the subject at the corresponding lead eQTL locus (pink: homozygous reference; green: heterozygous; blue homozygous Alternative). P-values and SNP position are given in Supp Table 6.

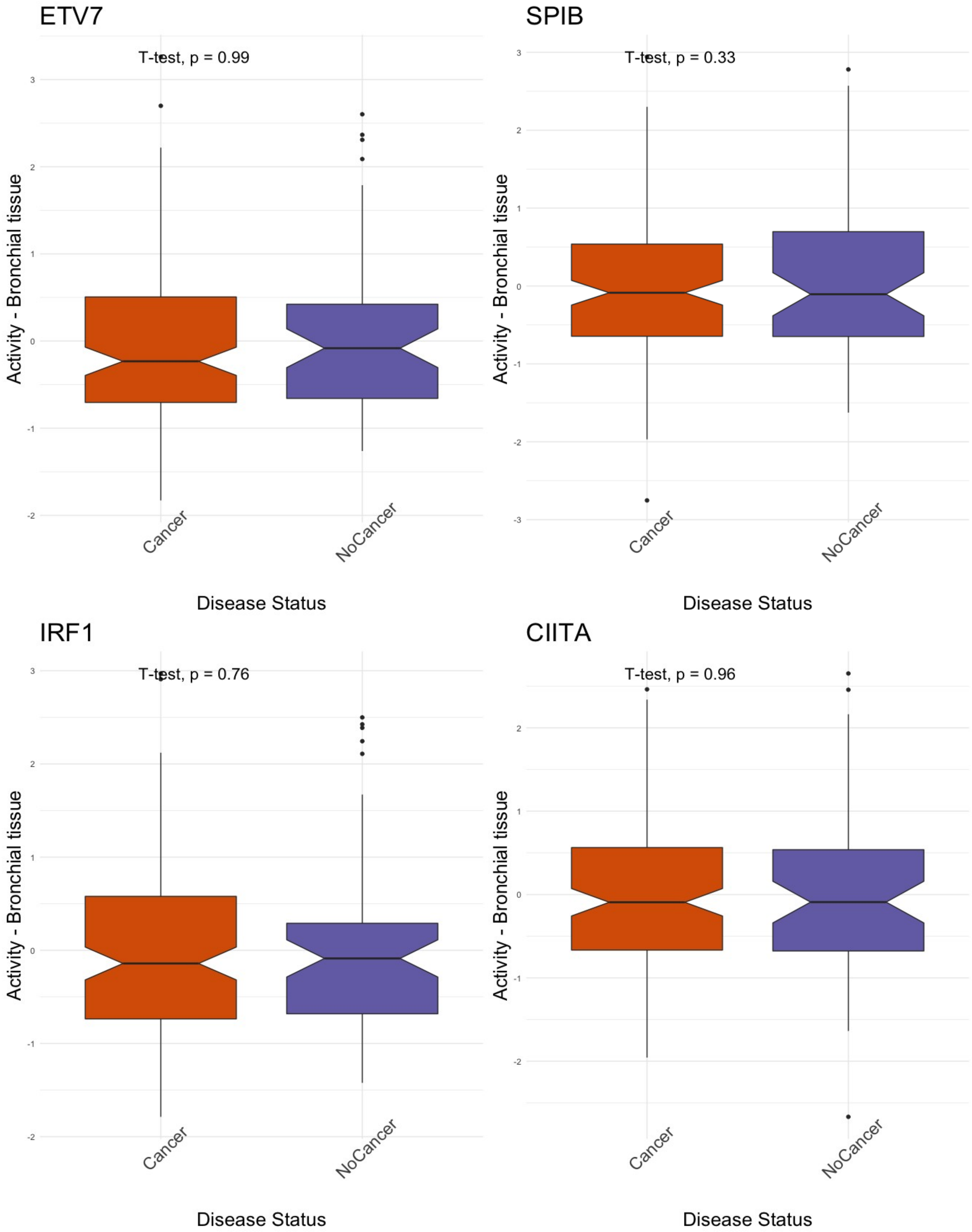

Fig. S15: (Equivalent to Fig 6b) The activity level of the 4 TFs that regulate a high number of risk and GWAS genes, but this time calculated for the Bronchial samples only, on a gene network that is inferred from the Bronchial samples. As in the nasal samples, we found no differences between clinic patients with and without cancer. We did not collect bronchial tissue from healthy volunteers for ethical reasons. Orange: cancer patients, purple: no cancer patients. P-values indicated at the top of each panel were calculated using a two sample t-test.

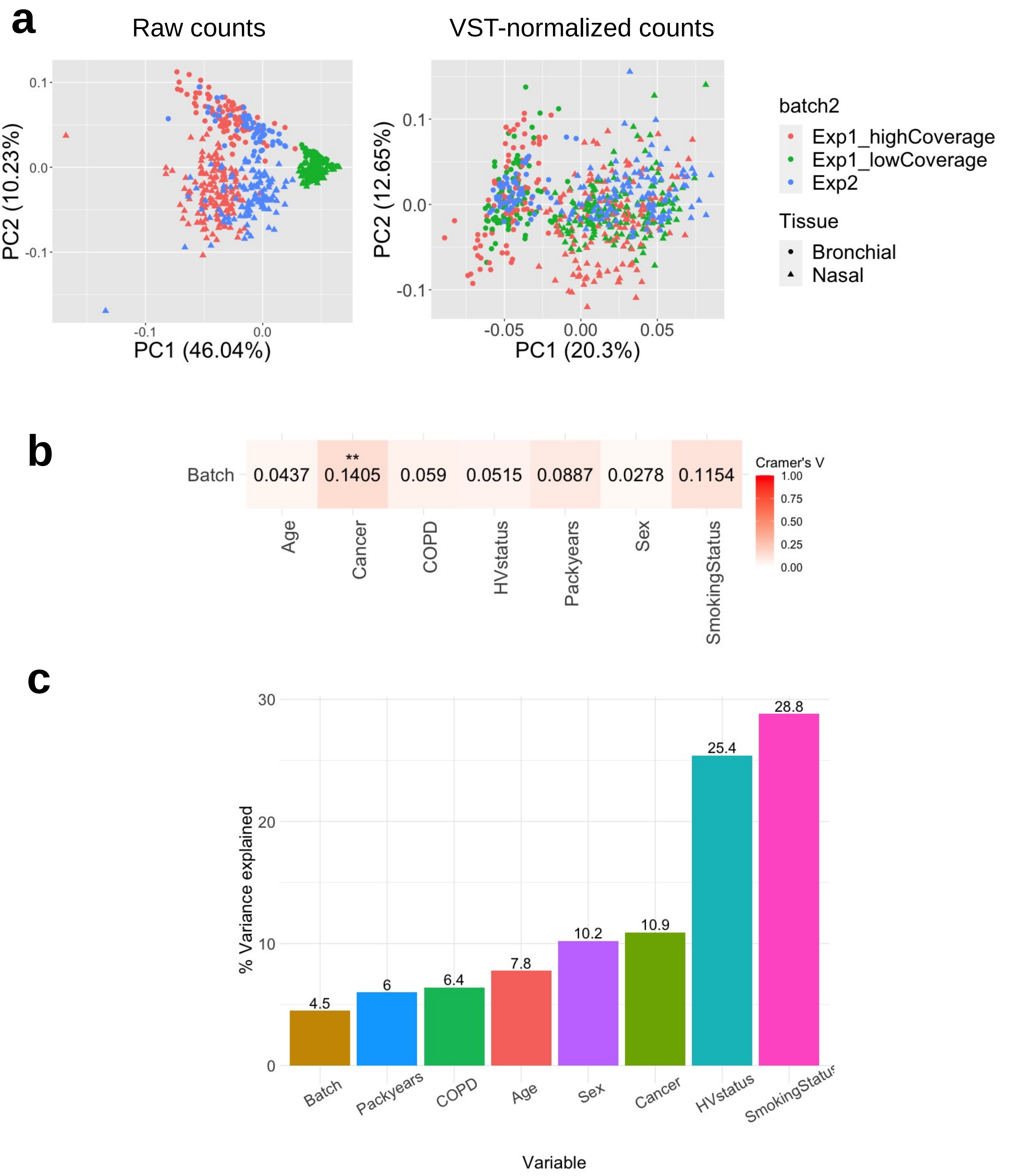

Fig. S16 Exploratory analysis. (**a)** PCA computed on all genes for every sample before (left) and after (right) VST-normalization. Each dot represent a sample, colored depending on the two experimental batches. Batch 2 is in blue, batch one was further stratified into 2 sets depending on the sequencing coverage (red: high coverage, green: low coverage). Shapes of the dot depend on the tissue of origin of the sample (triangle: nasal sample, circle: bronchial sample) **(b)** Strength and significance of association between experimental batch and clinical covariates; for each pair of covariates Cramer's V value (light red for values close to 0, dark red for values close to 1) and chi-square test pvalue are reported (*: P <= 0.05, **: P <= 0.01, ***: P <= 0.001). (c) Contribution of different clinical variables to the total explained variance in gene expression calculated using a random model on nasal samples.
